# Cesarean Section

**Published:** 1912-08

**Authors:** George Foy

**Affiliations:** Fellow of the Royal Society of Medicine, London; Surgeon to the Drumcowra Hospital, Dublin; Hon. Mem. of the Georgian State Medical Society, and of the Clarke County Medical Society, Ga.; Honorary Member of the Southern Surgical and Gynecological Association


### Cesarean Section.

GEORGE FOY, M.D., F.R.C.S.,

Fellow of the Royal Society of Medicine, London; Surgeon to the Drumcowra Hospital, Dublin; Hon. Mem. of the Georgian State Medical Society, and of the Clarke County Medical Society, Ga.; Honorary Member of the Southern Surgical and Gynecological Association.

HIDDEN away, out of the beaten track of tourists, in the noth-west corner of Ireland, lies the city of Clogher, on the banks of the Launy river, a tributary of the Blackwater. The city is of deep interest to both the archeologist and antiquarian and to all who take an interest in Irish history. The foundation dates further back than runneth the memory of man. On its site by the Launy the early Celts found the Cloch-Oir or Golden-Stone, which came to possess oracular reputation and was consulted by the Druids for centuries before the Chrisian era. By the Cloch-Oir the chiefs of Eviyol swore fealty to the O'Neils, the Princes of Ulster.

Hither Saint Patrick came and taught his Celtic audience that all knowledge, all wisdom, all love came from the Rock of Ages. His words bore fruit and in 444 his flock built him his first cathedral. The Druids had gone and the Golden Stone was forgotten; but the name Cloch-Oir remained associated with the place.

The city grew rapidly; the cathedral was rebuilt in 1041 and in 1295; it contained an Abbey and an Episcopal Palace, the former of which was richly endowed and the latter stood in its own extensive and well-wooded domain. But the Rebellion of 1641, was destructive of the prosperity of Clogher. All its beauties other than the old cathedral of Matthew MacCaitsaid and his great peal of sweet toned bells are decayed, and a short street of some thirty poor houses is all that remains of the prosperous cathedral city. In January 1738-9, a dweller in the city, one Mary Donally, an illiterate woman, who had acquired some reputation as a handywoman was called on to attend Alice O'Neal, aged 33 years, wife to a poor farmer near the village of Charlemont, and mother of several children, who took her labour pains; but who could not be delivered of her child by several women who attempted it. She remained in this condition twelve days; the child being judged to be dead after the third day. Mary Donally, on her arrival tried also to deliver her in the common way and her attempt not succeeding, performed the Cesarean section, by cutting with a razor first the containing parts of the abdomen and then the uterus, at the aperture of which she took out the child and secundines. The upper part of the incision was an inch higher, and to one side of the navel, and was continued about six inches downwards in the middle between the right osilium and the linea alba. She held the lips of the wounds together with her hand till one went a mile and returned with silk and the common needles which taylors use; with these she joined the lips in the manner of the stitch employed ordinarily for the *hare-lip,* and dressed the wound with whites of eggs, as she told Dr. Duncan Stewart, of Dungarsnon, (the original reporter of the case), some days after, when led by curiosity he visited the poor woman who had undergone the operation. The cure was completed with salves of Mary Donally's own compounding.

In about twenty-seven days the patient was able to walk about a mile on foot, and came to Doctor Duncan Stewart in a farmer's house where she showed him the wound covered with a cicatrice, where she complained of her belly hanging outwards on the right side, where he observed a tumour as large as a child's head; and she was distressed with the *Fluor Albus,* for which he gave her some medicines, and advised her to drink the decoctions of the vulnerary plants, and to support the side of her body with a bandage. The patient has enjoyed very good health ever since, manages her family affairs, and has frequently walked to market at Dungannou, which is six miles from her house.

Dr. Gabriel King, of Armagh, writing in 1740, refers thus to this case: "There is a woman living within five miles of this place (Armagh), from whom a midwife took a child by the *Cesarian* operation near two years ago. I saw the poor woman soon after and drew out the needles which the midwife had left to keep the lips of the wound together." And he adds: "This child was not extra-uterine for several besides the midwife assured me that a leg of it presented itself to view in the vagina before the operation."

The delivery of an extra-uterine foetus by the operation of laparotomy in which the operator was a butcher named O'Neil, which took place in the village of Prentrian, two miles from the city of Clogher, in 1640, is thus reported by the Rev. Dean Copping in the Philosophical Transactions:

"Sarah McKinna was married at the age of sixteen years. Ten months after her marriage, she found the symptoms of pregnancy and bore a child at the expiration of the usual time. Ten months after she was delivered of another; and each time had a speedy and easy delivery. Two months after her second delivery, symptoms of pregnancy appeared again, and increased in proportion to the time; but at the end of nine months those symptoms began to abate; and in a little time, she had no other reason for thinking she was with child, but the absolute stoppage of the catarmenia. Nor had she, during the space of six years and some months, any one return of them; but for the greatest part of that time, especially the first four years, she was perpetually afflicted with most violent pains in the middle region of the abdomen.

"Sometime in the event of her last pregnancy, which ended in such a usual manner, a swelling in her belly and other symptoms made her conclude she was again pregnant. About seven months after this uncertain account, a boil, as she thought, appeared about an inch and a half higher than her navel. During this time of her pregnancy she often found the symptoms of her being quick with child, till about six weeks before this boil, so she called it, appeared. It was attended with very great pain.

"She sent for one O'Neil, a butcher. This man came to her the Sunday after her message, and found her in an expiring condition. By this time the imposthumation had broken, and an elbow of the child had forced its way through, and appeared in view. At the request of herself and friends, he undertook to administer relief to her, and made so large an incision above and below the navel, as enabled him, by forcing his fingers under the jaw of the fœtus, to extract it; on which operation he met not with the least impediment. He afterwards looked into her belly, and seeing something black, he put in his hand, and extracted by pieces, a perfect skeleton of a child, and several pieces of black petrified flesh. After the operation, he stitched her up, and in six weeks she pursued her business about the house.

"She has been in good health ever after; only she had a navel rupture, owing to the ignorance of the man in not applying a proper bandage."

A similar case to this is recorded by Dr. Gabriel King, of Armagh, in Medical Essays, Edinburgh, 1742.

"The wife of a farmer, near Augher, fifteen miles from Armagh, who had borne some children, believed herself again with child in 1720. During the greatest part of the nine months of her pregnancy she was very sickly, but the labour pains did not come until her reckoning was out, at which time she had such midwives with her as the country affords, who, after endeavoring all they could, left her, and concluded she had no child to bear; the swelling of her belly diminished, and she became able to go about her ordinary business, though frequently she was sick and pained for about six years, when she again conceived. At the end of eight months, according to her reckoning, she felt extraordinary in the anterior part of her belly, and in a few days a small ulcer broke out below her navel; in some days more the elbow of a child appeared at this orifice. She brought out the whole arm with her bodkin, and got it cut off, but continued in great misery some days longer, but a footman to a gentleman in the neighborhood, and her relation, had the courage to pull out the remaining body of the child, while two other men, who went immediately after to the place and saw the child and cavity from which it was brought, assured me it was a full and complete child except the arm which had been taken away before. When I went, about three weeks after to see this poor woman, she was extremely emaciated, and the wound was almost closed. Upon probing at a little distance toward the left side from where the wound was I felt small bones under my fingers, which seemed to be contained in a bag so thin that I was persuaded that it might have been cut when the other child was extracted, and these bones been brought out at the same orifice. She showed me some decayed bones, which had evidently belonged to a human fœtus, that she had passed partly by stool and partly with her urine, as she informed me, and they were daily coming away. She believed she would die, but after several visits, I found her walking out in the fields, and she has lived seven years since."

These three remarkable cases occurred in the Clogher Valley district and within a short distance of each other; a country in which the women are noted for their power of thwarting pain and their great determination.

